# Failure of Sequential JADA® Devices in Major Postpartum Hemorrhage Requiring Emergent Supracervical Hysterectomy: A Case Report

**DOI:** 10.7759/cureus.104498

**Published:** 2026-03-01

**Authors:** Salah Riyadh, Sammy Jaber, Joseph Boujaoude, Jessica Branham

**Affiliations:** 1 Department of Obstetrics and Gynecology, Pikeville Medical Center, Pikeville, USA; 2 Kentucky College of Osteopathic Medicine, University of Pikeville, Pikeville, USA

**Keywords:** emergency obstetric hysterectomy, jada device, postpartum hemorrhage, supracervical hysterectomy, uterine atony

## Abstract

Major postpartum hemorrhage (PPH) continues to be a significant contributor to maternal health complications, and rapid control of bleeding is essential to prevent hemodynamic instability and the need for surgical intervention. The JADA^®^ uterine vacuum system has been increasingly adopted as a primary tool to manage PPH caused by uterine atony. Although reported success rates are high, particularly when used early, device failure can still occur and requires timely escalation of care. We present the case of a 30-year-old woman, gravida 3 para 2, who developed major PPH following a spontaneous vaginal delivery at term. Her bleeding was attributed to uterine atony and persisted despite the administration of multiple uterotonic agents, including Pitocin, methylergonovine, Hemabate, rectal misoprostol, and tranexamic acid. A JADA^®^ device was placed but did not achieve adequate hemorrhage control. A second JADA^®^ device was subsequently inserted, yet bleeding continued. The patient required transfusion of packed red blood cells and was taken emergently to the operating room for a supracervical hysterectomy, after which her condition stabilized. This case demonstrates sequential JADA^®^ device failure in the setting of major PPH and emphasizes the need for clinicians to promptly recognize insufficient response, understand the limitations of uterine vacuum systems, and be prepared to escalate to surgical management when bleeding persists. As the JADA^®^ device becomes further integrated into PPH algorithms, documenting real-world experiences with device failure is essential for refining clinical expectations, improving patient care, and reducing mortality.

## Introduction

Postpartum hemorrhage (PPH) remains a leading cause of maternal morbidity and mortality, highlighting the need for rapid identification and intervention to prevent hemodynamic collapse [1]. PPH is defined as cumulative blood loss exceeding 1,000 mL or blood loss accompanied by signs of hypovolemia, regardless of the route of delivery. It can result from retained placental tissue, lacerations, bleeding disorders, or most commonly, uterine atony, which is the failure of the myometrium to contract adequately following placental delivery [2]. When standard pharmacologic uterotonics such as oxytocin, methylergonovine, and carboprost fail to achieve hemostasis, clinicians face critical, time-sensitive decisions regarding escalation to mechanical or surgical management [1].

Intrauterine vacuum-assisted hemorrhage-control devices, such as the JADA^®^ device, received FDA clearance in August 2020 and have been increasingly incorporated into PPH management algorithms. The JADA^®^ system is among the most commonly used vacuum-assisted devices, with a reported success rate of 94% for controlling postpartum bleeding within a median time of three minutes, emerging as an alternative mechanical strategy for the management of uterine atony [3]. Unlike balloon tamponade systems, which rely on outward pressure against the uterine wall, vacuum-assisted devices apply low-level negative pressure within the uterine cavity to promote myometrial apposition and physiologic uterine contraction, thereby reducing bleeding from the uterine vasculature [4]. Although clinical trials such as the PEARLE study and subsequent observational data have demonstrated high rates of hemorrhage control with vacuum-based systems, device failure may still occur in approximately 6% of cases, particularly in instances of severe or rapidly evolving hemorrhage [4]. Cases of PPH refractory to vacuum-assisted hemorrhage control remain poorly characterized and present a significant clinical challenge, requiring providers to balance fertility-preserving interventions with timely escalation to surgical management in the setting of ongoing massive blood loss.

This case report details the clinical course of a 30-year-old multigravida who experienced major PPH following a spontaneous vaginal delivery. Despite administration of multiple uterotonic agents and the sequential placement of two separate JADA^®^ devices, hemorrhage persisted. The following sections describe the failure of these mechanical interventions and the subsequent decision to perform an emergent supracervical hysterectomy to achieve definitive hemostasis. This report underscores the importance of recognizing the limitations of vacuum-assisted devices and the necessity of prompt surgical escalation in refractory cases of PPH.

## Case presentation

A 30-year-old gravida 3 para 2 woman at 37 3/7 weeks’ gestation was admitted for induction of labor but was noted to be in early spontaneous labor upon arrival. She received epidural anesthesia for pain management and oxytocin for labor augmentation. She progressed to complete cervical dilation and delivered a live female infant via spontaneous vaginal delivery at 19:06, weighing 3,160 g. Delivery occurred in the right occiput anterior position without nuchal cord or shoulder dystocia. Delayed cord clamping was performed, and the placenta was delivered spontaneously.

Placental inspection demonstrated an intact placenta with a three-vessel umbilical cord. The uterus was cleared of clots, and vaginal examination revealed an intact perineum without evidence of laceration. Intravenous oxytocin was initiated; however, uterine atony was suspected, and bimanual uterine massage was performed in addition to administration of 800 mcg of misoprostol per rectum. Despite transient uterine firmness, recurrent episodes of brisk vaginal bleeding persisted.

The uterus was explored, and additional clots were removed. A first intrauterine vacuum-assisted hemorrhage-control device was placed; however, despite appropriate placement and activation, the device failed to evacuate blood or produce meaningful improvement in uterine tone and was therefore removed. Given ongoing hemorrhage, a second vacuum-assisted intrauterine hemorrhage-control device was placed. Following activation of the second device, approximately 500 mL of blood was evacuated into the device canister; however, sustained uterine tone was not achieved, and hemorrhage continued.

Persistent bleeding prompted further pharmacologic therapy, including methylergonovine 0.2 mg intramuscularly, carboprost tromethamine 0.25 mg intramuscularly, and tranexamic acid 1 g intravenously. A Bakri balloon was then placed intrauterinely without difficulty; however, balloon inflation was unsuccessful, and the device was removed. The patient continued to experience heavy vaginal bleeding with recurrent uterine atony despite these measures.

During this period, the patient developed clinical signs of hemodynamic instability, including near-syncope and transient loss of consciousness. Two units of packed red blood cells were administered emergently, and additional large-bore intravenous access was obtained. The interventional radiology team was consulted; however, given the patient’s worsening hemodynamic instability and concern that further delay would pose significant risk, the decision was made to proceed directly to the operating room for definitive surgical management.

Following failure of pharmacologic therapy and mechanical hemorrhage-control measures, the patient underwent a supracervical hysterectomy for definitive hemorrhage control. Intraoperatively, uterine atony was confirmed, and no alternative surgical sources of bleeding were identified. Hemostasis was achieved following hysterectomy, and the patient’s condition stabilized.

Delivery occurred at 19:06 on hospital day 1. Serial laboratory evaluations during the postpartum course demonstrated progressive anemia consistent with ongoing hemorrhage (Table 1). Hemoglobin declined from 11.0 g/dL at 23:29 on day 1 to 10.6 g/dL by 23:34 on day 2, with a nadir of 7.6 g/dL at 05:36 on day 3 prior to partial recovery. Hematocrit values followed a similar downward trend. Coagulation studies obtained during this period demonstrated preserved hemostatic function, with prothrombin time and activated partial thromboplastin time within normal limits and fibrinogen levels remaining elevated (421-437 mg/dL), findings not suggestive of consumptive coagulopathy. These laboratory trends supported acute blood loss secondary to uterine atony rather than a primary coagulopathic process.

**Table 1:** Complete blood count with differential Serial complete blood counts with differential and coagulation studies obtained following PPH demonstrate physiologic leukocytosis and progressive anemia over a three-day period. Asterisks (^*^) indicate values outside the reference range. FEU, fibrinogen equivalent unit; PPH, postpartum hemorrhage

Parameter	Units	Day 1 (23:29)	Day 2 (20:50)	Day 2 (23:34)	Day 3 (05:36)	Day 3 (06:54)	Reference range
White blood cells (auto)	× 10³/µL	12.9^*^	16.1^*^	21.8^*^	13.4^*^	-	4.5-11.0 × 10³/µL
Hemoglobin	g/dL	11.0^*^	10.9^*^	10.6^*^	7.6^*^	8.5^*^	12.0-16.0 g/dL
Hematocrit	%	32.4^*^	31.6^*^	31.7^*^	23.1^*^	24.7^*^	36-48%
Platelets (auto)	× 10³/µL	288	227	246	149	-	150-400 × 10³/µL
Neutrophils (manual)	%	75	-	-	81^*^	-	50-70%
Lymphocytes (manual)	%	16^*^	-	-	13^*^	-	18-42%
Monocytes (manual)	%	7	-	-	5	-	2-11%
Eosinophils (manual)	%	1	-	-	1	-	1-3%
Prothrombin time	seconds	-	-	12.3	-	12.7	11.5-14.5 seconds
International normalized ratio	-	-	1.08	1.09	-	1.12	0.9-1.2
Activated partial thromboplastin time	seconds	-	25	25	-	25	25-35 seconds
Fibrinogen	mg/dL	-	421^*^	437^*^	-	430^*^	200-400 mg/dL
D-dimer (FEU)	ng/mL	-	4,477^*^	-	-	-	<500 ng/mL

Postoperatively, the patient recovered without further hemorrhagic complications. Hemodynamic parameters stabilized, transfusion requirements ceased, and she demonstrated appropriate postoperative recovery prior to discharge. Informed consent for publication of this case was obtained from the patient.

## Discussion

PPH remains a leading cause of maternal morbidity and mortality worldwide and continues to represent a major obstetric emergency even in high-resource settings [5]. Despite advances in obstetric care, the incidence of PPH has increased over the past two decades, with a substantial proportion of affected patients requiring blood transfusion or invasive intervention [6]. Uterine atony is the most common cause of PPH and results from inadequate myometrial contraction following placental separation, allowing continued bleeding from the uterine vasculature [7]. At the time of management in our patient, other PPH etiologies were rapidly excluded, and uterine atony was determined to be the cause in the absence of trauma, retained tissue, or coagulopathy.

Management of PPH follows a stepwise escalation strategy beginning with uterine massage, bimanual compression, and uterotonic agents, including oxytocin, methylergonovine, carboprost, misoprostol, and tranexamic acid [7,8]. Concurrent with pharmacologic therapy, mechanical interventions such as intrauterine balloon tamponade or vacuum-assisted hemorrhage-control devices may be employed before escalation to surgical management [8]. Balloon tamponade devices achieve hemostasis through outward pressure against the uterine wall, whereas vacuum-assisted systems apply low-level negative pressure within the uterine cavity to promote uterine contraction [4]. Vacuum-assisted intrauterine hemorrhage-control devices are a more recent innovation and have been increasingly incorporated into institutional PPH algorithms. As their clinical use expands, recent literature emphasizes the need to better characterize and address knowledge gaps related to device failure [5].

To our knowledge, no similar case reports describing sequential device failure exist in the current literature, limiting guidance on escalation strategies when mechanical hemorrhage control is ineffective. Current clinical guidelines provide limited direction on identifying inadequate device response or determining optimal timing for escalation, placing significant reliance on clinician judgment in time-sensitive situations [5,7]. Recognition of ongoing hemorrhage is further complicated by the known inaccuracy of visual estimation of blood loss, which frequently underestimates true volume, particularly at higher levels [5]. Delayed recognition of ineffective hemorrhage-control measures may result in increased transfusion requirements and higher risk of maternal morbidity. Early identification of insufficient response to mechanical interventions is therefore critical to minimizing adverse outcomes.

The present case illustrates, to our knowledge, the first documented instance of sequential failure of vacuum-assisted intrauterine hemorrhage-control devices in the setting of major PPH due to uterine atony. Despite appropriate pharmacologic and mechanical interventions, bleeding persisted, necessitating blood transfusion. After all available non-surgical, fertility-preserving interventions were attempted, the decision to escalate to surgical management via supracervical hysterectomy was made to achieve definitive control of hemorrhage and stabilize the patient. This case underscores the importance of maintaining a low threshold for escalation when hemorrhage continues despite device placement and highlights the limitations of mechanical adjuncts in severe PPH.

Further research is needed to better define predictors of vacuum-assisted device failure, establish objective markers of inadequate response, and develop standardized escalation guidelines for refractory PPH [5,9]. Although predictors of device failure are under investigation, they are not yet well established. Our patient had no additional risk factors beyond obesity, yet her PPH proved refractory to all mechanical interventions attempted [10]. Improved evidence-based protocols may facilitate earlier definitive intervention and reduce transfusion-related morbidity in patients experiencing severe PPH.

## Conclusions

This case describes the failure of sequential vacuum-assisted intrauterine hemorrhage-control devices in managing major PPH secondary to uterine atony, ultimately requiring emergent supracervical hysterectomy. In this single-patient experience, escalation from uterotonics to mechanical interventions, including placement of two separate devices, did not achieve sustained hemostasis despite initial attempts at vacuum-mediated blood evacuation. Progressive hemodynamic instability and ongoing hemorrhage necessitated definitive surgical management.

Although vacuum-assisted devices are increasingly incorporated into PPH management algorithms, this case highlights that device failure can occur even with appropriate placement and escalation. The clinical course underscores the importance of continuous reassessment, prompt recognition of inadequate response, and timely transition to surgical intervention when bleeding persists. As an individual case observation, these findings are hypothesis-generating and contribute to real-world clinical understanding rather than establishing practice-changing recommendations. Further studies are needed to better characterize predictors of device nonresponse and to refine decision-making thresholds for escalation.
